# 

**Published:** 1846-10

**Authors:** 


*Obituary Record.*—Died, in London, Aug. 6th, of an attack of Cholera, John Bostock, M. D., F. R. S., at the age of 73. He was well known in this country by his valuable Elements of Physiology and other writings.

——, in Paris, in the 36th year of his age, Dr. Thibert, the inventor of very beautiful artificial anatomical and pathological models in relief.

——, at Cambridge, Mass., Benjamin Waterhouse, M. D., aged 92. Dr. W. was formerly Professor of Theory and Practice in Harvard University.

---

*The Annalist.*—We have received the first number of this new medical periodical. It is a bi-weekly of 24 pages, for $2,00 per annum. Wm. C. Roberts, M. D., well known as a writer and practitioner, is the editor. We heartily wish it success.

---

*Books received.*—The following books lately published by Barrington and Haswell, Philadelphia, have been received too late for farther notice in this number. T. and M. Butler will receive orders for them. We shall speak of their merits in our next number.

Fox and Harris on the Teeth.
Gross' Pathological Anatomy, new edition.
Gross' Liston's Surgery.
Gerhard on the Chest.
Neill on the Arteries.
Neill on the Nerves.
Ludlow's Examinations.
Bell and Stokes' Practice.
Dublin Quarterly Journal.

Our usual exchanges are hereby acknowledged.

